# Dengue infection changes the expressions of CD154 and CD148 in human platelets

**DOI:** 10.1016/j.virusres.2024.199519

**Published:** 2024-12-26

**Authors:** Sayali Vedpathak, Sonali Palkar, AkhileshChandra Mishra, Vidya A Arankalle, Shubham Shrivastava

**Affiliations:** a Department of Translational Virology, Interactive Research School for Health Affairs (IRSHA), Bharati Vidyapeeth (Deemed to be University), Katraj-Dhankawadi, Pune 411043, India; b Department of Community Medicine, Bharati Vidyapeeth (Deemed to be University) Medical College and Hospital, Katraj-Dhankawadi, Pune 411043, India

**Keywords:** Dengue infection, Platelets, CD154, CD148, CD41/CD61

## Abstract

Platelets are essential for hemostasis and vascular integrity. Platelets recognize dengue virus through the DC-SIGN receptor. Upon pathogen recognition, platelets rapidly modulate the expression of adhesion molecules to trigger immune cell interactions and regulate the immune response. In this study, we aimed to examine the expression levels of three molecules, CD151, CD154, and CD148 on platelets in dengue patients. A significantly increased expression of CD154 and reduced expression of CD148 was observed in dengue patients compared to healthy subjects (*p* < 0.0001). Moreover, a strong positive correlation between CD148 and CD41/CD61 (*p* < 0.0001) was noted in dengue patients. In summary, we demonstrated the altered expressions of CD154 and CD148 on platelets in dengue patients that may play a crucial role in dengue pathogenesis.

## Highlights

• An increased expression of membrane-associated CD154 on platelets may enhance inflammation in dengue patients.
• Reduced levels of membrane-associated CD148 on platelets were observed in dengue patients.
• Strong correlation of CD148 with αIIbβ3 (CD41/CD61) was noted in dengue patients.

## 1 Introduction

Dengue is the most common tropical viral disease. Since the start of 2023, there has been a significant rise in dengue cases, with over 5000 dengue-related deaths reported in over 80 countries (World Health Organization 2023). Platelets are the major cell population affected by the dengue virus and are highly activated in dengue patients (Hottz et al., 2014; Ojha et al., 2017; Vedpathak et al., 2024). Platelets are known to regulate innate immune response, hemostasis, and thrombosis. Besides this, platelets also contribute to inflammation, host defense, and adhesion to endothelium to regulate vasculature (Vieira-de-Abreu et al., 2012; Janus-Bell and Mangin, 2023). Platelets recognize pathogens through pathogen-associated molecular patterns (PAMPs) or damage-associated molecular patterns (DAMPs) by binding to receptors such as Toll-like receptors (TLRs), C-type lectin receptors (CLRs), and NOD-like receptors (NLRs) resulting in platelet activation, secretion of granules, and interaction with leukocytes to trigger both the innate and adaptive immune response (Dib et al., 2020 Oct). In the case of dengue virus (DENV) infections, platelets recognize DENV through DC-SIGN receptors (Simon et al., 2015 Jul 16).

Platelets express several adhesion molecules including αIIbβ3 (CD41/CD61), glycoprotein (GP)VI, α5β1, P-selectin (CD62p), CD40 L (CD154), CD151, and CD148 (PTPRJ) on their surface (Vieira-de-Abreu et al., 2012; Janus-Bell and Mangin, 2023). The interaction of platelet adhesion receptors with the specific ligands on immune cells or endothelium is crucial for maintaining thrombi at the site of vascular injury. One of the most commonly found integrins, αIIbβ3 facilitates platelet spreading and subsequent aggregation through the binding to VWF and fibrinogen during injury (Hosseini et al., 2019 Oct 23). GPVI, a receptor for collagen and laminin, and α5β1, a receptor for fibronectin also contribute to platelet adhesion on extracellular matrix proteins. This process is essential to maintain hemostasis and thrombosis at the sites of vascular injury (Boulaftali et al., 2018). Upon vascular injury, P-selectin, an adhesion molecule exposed on the surface of activated platelets, promotes interactions with leukocytes through the binding of P-selectin glycoprotein ligand 1 (PSGL1) and secretion of chemokines to assist in the recruitment of monocytes, neutrophils, and lymphocytes to the inflamed endothelium. Interaction of platelets with immune cells and endothelium resulted in platelet-leukocyte aggregate (PLA) formation, secretion of inflammatory cytokines, and thrombus formation (Ghasemzadeh and Hosseini, 2013 Mar). Platelet leukocyte crosstalk in the presence of chemokines, and cytokines induced the activation of hyper-inflammatory monocytes resulting in cytokine storm, and neutrophil extracellular traps formation, contributing to thrombosis and inflammation. Increased levels of PLA were reported in HIV, influenza, and COVID-19 patients (Taus et al., 2020 Dec). The increased levels of platelet-monocyte aggregates were also reported in dengue patients (Hottz et al., 2014).

Activated platelets stimulate an inflammatory response through the CD40L(CD154)-CD40 signaling pathway (Chakrabarti et al., 2007; Cognasse et al., 2022; Duffau et al., 2010). CD154 on activated platelets interacts with CD40 ligand expressed on endothelial cells and induces endothelial cells to secrete inflammatory signals to recruit leukocytes at the site of injury and promote inflammation (Henn et al., 1998). CD151 is a major component of hemidesmosome and is regulated by α6β4 integrin. Hemidesmosomes are specialized junctional complexes that allow the binding of cells to the basement membrane and therefore, CD151 regulates cell adhesion processes (Sterk et al., 2000 May 15). CD148 is essential for GPVI-mediated inside-out adhesion signaling in platelets. CD148 dephosphorylates the activation loop tyrosine and promotes optimal platelet adhesion and spreading on collagen (Ellison et al., 2010 Jul). Platelet adhesion to injured vasculature is important to maintain thrombus formation and impairment in this process may lead to bleeding manifestations in dengue patients. The interplay of several molecules is vital in platelet signaling during dengue infection. However, the presence and functionality of CD151, CD154, and CD148 on platelets have not been studied extensively in dengue infection.

## 2 Methods

### 2.1 Study subjects

On approval by the Institutional Human Ethics Committees of Bharati Vidyapeeth Medical College & Hospital, Pune, a total of 90 subjects were recruited for this study, including 19 healthy subjects and 71 dengue patients. Blood samples were collected in acid-citrate dextrose (ACD) tubes. Dengue infection was confirmed through ELISAs by the presence of NS1 antigen and/or anti-dengue IgM antibodies. Secondary dengue infection was confirmed by high-titer anti-dengue IgG antibodies.

### 2.2 Flow cytometry

For platelet separation, blood samples were centrifuged at 400 g for 10 min. Platelet-rich plasma (PRP) was collected and centrifuged at 2000 g for 10 min. Isolated platelets were resuspended in Dulbecco's Phosphate Buffered Saline (DPBS). Platelets were stained with the following, fluorescent-labeled antibodies BV510-CD45, PerCP-Cy5.5-CD41/CD61, BV785-CD62P, APC—CD151, PE-CY7-CD154, and PE-CD148 and incubated at 4 °C for 30 min. After incubation, the platelets were washed once with DPBS, and cells were acquired with CytoFlex LX (Beckman Coulter, USA) to check the expression of surface receptors on platelets. 10,000 total gated events were recorded for each sample.

### 2.3 Statistical analysis

All data were represented as mean ± standard error. Statistical analysis was carried out using a non-parametric Mann-Whitney test to compare the two groups. To find the association between CD41/CD61 and CD148 in dengue patients, a non-parametric Spearman correlation regression test with a 95 % interval was performed using GraphPad Prism software (version 10.0.3).

## 3 Results

The clinical characteristics of 19 healthy subjects and 71 serologically confirmed dengue patients were presented in Table 1. In our study groups, the median age of healthy subjects and dengue patients were similar, 23 and 28 respectively. It has been reported that dengue patients at older ages were more likely to develop symptomatic dengue than younger individuals for both primary and secondary infections (Thai et al., 2011 Jun). Older dengue patients aged ≥ 60 years were at higher risk of developing DHF than adult dengue patients (Chhong et al., 2020 Sep 1). We also noticed that significantly higher numbers of males were infected with the dengue virus (47/71, 66.2 %) than females (24/71, 33.8 %, *p* < 0.001). We reported male predominance among dengue patients similar to other dengue-endemic countries, Sri Lanka, Philippines, Singapore, and Lao People's Democratic Republic (Anker and Arima, 2011 Jun 30; Prasith et al., 2013 May 21). Of 71 dengue patients, 5 (7 %) patients were positive only for NS1 antigen, 34 (48 %) patients were positive for both NS1 and anti-dengue IgM antibody, and 32 (45 %) were positive only for anti-dengue IgM antibody. 17 patients had primary dengue and 54 patients had secondary dengue infection. As per WHO (2009) guidelines, 22 patients were categorized as mild dengue without warning signs (WS-), 44 patients as dengue with warning signs (WS+), and 5 patients in severe dengue categories (SD). Of 71 dengue patients, 63 patients had thrombocytopenia with a platelet count below 1,50,000/µL. Of these, 32 patients had severe thrombocytopenia with a platelet count below 50,000/µL.

**Table 1:** Clinical features of recruited subjects.

Clinical features of recruited subjects*		Healthy subjects (*n* = 19)			Dengue patients (*n* = 71)		
Median Age, (range in years)		23 (20–39)			28 (2–76)		
Gender							
Female		13			24		
Male		6			47		
Dengue confirmatory tests (%)							
NS1+		0			5 (7)		
NS1+ IgM+		0			34 (48)		
IgM+		0			32 (45)		
Primary Dengue		N.A.			17 (24)		
Secondary Dengue		N.A.			54 (76)		
Platelet count of dengue patients	**Total Dengue patients**	**WS-**	**WS+**	**SD**	**p-value**@	**p-value**#	**p-value^$^**
Number of subjects	71	22	44	5			
Median Platelet count per µL (Interquartile range)	56,000 (27,000–93,000)	91,500 (71,750–1,32,000)	35,000 (20,250–79,750)	31,000 (19,500–46,000)	**<0.0001**	**<0.0001**	0.5685
Number of patients with a platelet count below 50,000 per µL	32	0	28	4	**<0.0001**	**0.0001**	0.816

⁎ Data are expressed as numbers or else specified. WS- represents dengue patients without warning signs, WS+ represents dengue patients with warning signs, SD denotes severe dengue patients, N.A. denotes not applicable

@ indicates statistical significance on comparison of WS- with WS+ patients

# indicates statistical significance on comparison of WS- with SD patients, and ^$^indicates statistical significance on comparison of WS+ with SD patients. Data were analyzed by non-parametric Mann-Whitney test.

**Fig. 1:**
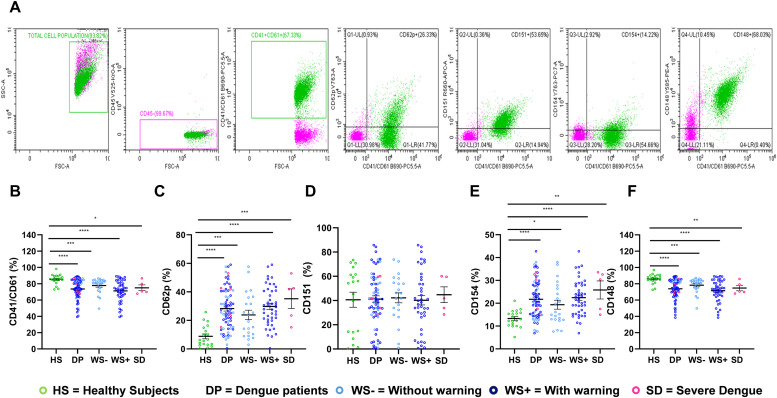
Alteration in the expression of platelet adhesion molecules in dengue patients with different clinical presentations. (A) Gating strategy of platelet surface receptor. Expression of (B) CD41/CD61, (C) CD62p (D) CD151 (E) CD154, and (F) CD148 on platelet surface of healthy subjects (*n* = 19) and dengue patients with different clinical presentations, WS- (*n* = 22), WS+ (*n* = 44), and SD (*n* = 5). HS represents healthy subjects, DP represents dengue patients, WS- denotes dengue patients without warning signs, WS+ denotes dengue patients with warning signs and SD denotes severe dengue patients. Data was represented as mean ± standard error. Clinical data was analyzed by a non-parametric Mann-Whitney test in which * represents *p* < 0.05, ** represents *p* < 0.01, *** represents *p* < 0.001 and **** represents *p* < 0.0001.

We first examined the changes in the expressions of adhesion molecules on the platelets in response to dengue infection. Fig. 1A represents the gating strategy used for flow-cytometric analysis. Briefly, CD45 enriched leukocyte negative (>99 %) cell population was selected and then CD41/CD61 was plotted to obtain the platelet population. CD62p (platelet activation), CD148, CD151, and CD154 expression on platelets were plotted against the CD41/CD61 positive platelet population. As reported earlier (Vedpathak et al., 2024), we observed significantly reduced expression levels of CD41/CD61 in dengue patients (*p* < 0.0001) including WS- patients (*p* = 0.0005), WS+ patients (*p* < 0.0001), and SD patients (*p* = 0.01) in comparison to healthy subjects (Fig. 1B). Platelet activation was seen with the increased expression of CD62p on platelets in all dengue patients (*p* < 0.0001), WS- (*p* = 0.0004), WS+ (*p* < 0.0001) and SD patients (*p* = 0.0009) as compared to healthy subjects (Fig. 1C).

The other three platelet-specific adhesion molecules had differential expressions in dengue patients. Expression levels of CD151 remained unchanged in dengue patients irrespective of the dengue disease category when compared to healthy subjects (Fig. 1D). We noted an increased expression of CD154 in dengue patients (*p* < 0.0001) as compared to healthy subjects. Expression levels of CD154 were significantly higher in WS- (*p* = 0.04), WS+ (*p* < 0.0001), and SD patients (*p* = 0.003) as compared to the healthy subjects (Fig. 1E). Although there was an increasing trend of CD154 expression levels with increasing disease severity, no significant difference was seen in WS+ & SD patients as compared to WS- dengue patients, probably due to a smaller sample size. On the contrary, in comparison to healthy subjects, reduced levels of expressions of CD148 were seen in all dengue patients (*p* < 0.0001), WS- (*p* = 0.0006), WS+ (*p* < 0.0001) and SD patients (*p* = 0.005) (Fig. 1F). No significant difference in CD148 expression was noted in WS+ & SD patients compared to WS- patients. No significant difference was observed in the expression levels of CD41/CD61 (*p* = 0.21); CD62p (*p* = 0.17); CD151 (*p* = 0.14); CD154 (*p* = 0.16) and CD148 (*p* = 0.19) between primary and secondary dengue patients.

**Fig. 2:**
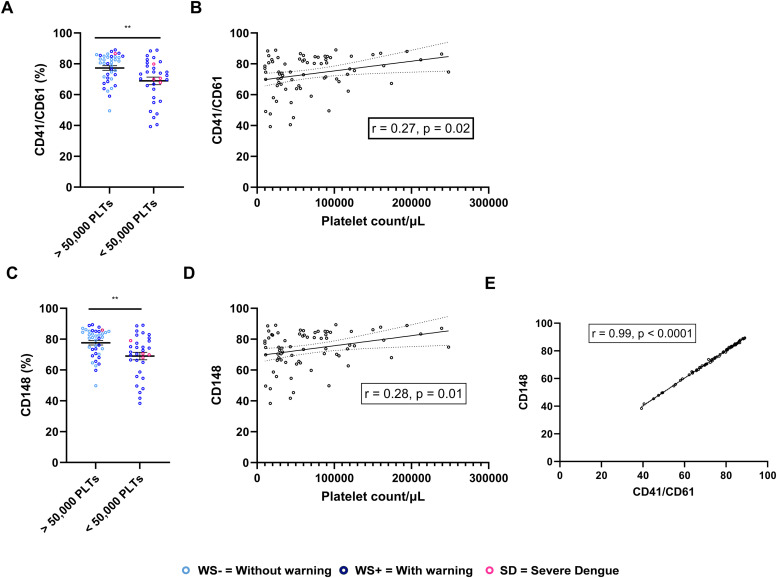
CD41/CD61 and CD148 correlate with decreased platelet count in dengue patients. (A) Comparison of expression of CD41/CD61 between dengue patients with platelet count >50,000/μL and platelet count <50,000/μL. (B) Correlation analyses of platelet count with CD41/CD61 platelet surface expression. (C) Comparison of expression levels of CD148 in dengue patients having platelet count >50,000/μL and <50,000/μL. (D) Correlation analyses of thrombocytopenia with CD148 surface expression on platelets. (E) Correlation analysis between expression of CD41/CD61 and CD148 on platelets of dengue patients. Data is represented as mean ± standard error. Data was analyzed by a non-parametric Mann-Whitney test. Correlation analyses were performed using a two-tailed Spearman's correlation coefficient test. ** represents *p* < 0.01.

As thrombocytopenia is an important determinant of disease severity in dengue patients, we examined the association of expression levels of adhesion molecules with platelet count in the dengue patients. CD41/CD61 levels were significantly reduced in the dengue patients with a platelet count <50,000/µl than in dengue patients with a platelet count >50,000/µl (*p* = 0.005, Fig. 2A). Similarly, we noted a weak but positive correlation of CD41/CD61 with the platelet count in dengue patients (*p* = 0.02, *r* = 0.27, Fig. 2B). Notably, in the dengue patients with a platelet count <50,000/µl, the expression of CD148 was significantly reduced when compared to patients with a platelet count >50,000/µl (*p* = 0.004, Fig. 2C). We noted a weak positive correlation of CD148 with the platelet count in dengue patients (*p* = 0.01, *r* = 0.28, Fig. 2D). As both the surface markers were similarly affected due to decreased platelet count, we measured the correlation between CD41/CD61 and CD148. Strikingly, a strong positive association was observed between CD41/CD61 and CD148 (*p* < 0.0001, *r* = 0.99, Fig. 2E).

## 4 Discussion

This study was the first to report the altered expressions of CD154 and CD148 on activated platelets in dengue patients. We and several other groups have reported higher platelet activation with increased disease severity in dengue patients (Hottz et al., 2014; Ojha et al., 2017; Vedpathak et al., 2024). We reported reduced expression of CD41/CD61 (αIIbβ3) in dengue patients as observed in patients with Glanzmann thrombasthenia and leptospirosis. Reduced expression of αIIbβ3 abolished the platelet aggregation ability in patients with Glanzmann thrombasthenia (Kannan et al., 2003 Oct). Whereas a decreased level of αIIbβ3 impedes the binding of platelets to fibrinogen and increases the bleeding manifestations in patients with leptospirosis (Tunjungputri et al., 2017 Sep 21). Moreover, an *in-vitro* study indicated that platelets produced from DENV-infected MEG-01 cells had a low level of αIIbβ3 with compromised aggregation ability of platelets (Banerjee et al., 2020 Nov 11). Altogether, it suggests that loss of CD41/61 receptors hampers the aggregation and thrombus formation ability of platelets and might result in bleeding manifestations during dengue infection. Downregulation of platelet adhesion receptors is a controlling mechanism of thrombosis as shown with stored platelets (Hosseini et al., 2019 Oct 23). Platelets lose the expression and function of adhesion receptors, GPIbα, and GPVI due to ectodomain shedding and micro vesiculation during storage. An alternative mechanism of reduced expression of αIIbβ3 was linked with calpain-mediated cleavage of the cytoplasmic part of β3 in stored platelets that may control the adhesive function of αIIbβ3, aggregation, and thrombus formation during platelet storage (Hosseini et al., 2019 Oct 23).

Platelet activation during viral infection is monitored by platelet-leukocyte aggregation, increased expression of P-selectin (CD62p), and increased PAC-1 binding to platelets (Hottz et al., 2014; Ojha et al., 2017; Zhang et al., 2020 Sep 4). Higher CD62p levels in dengue patients emphasize the platelet activation during dengue infection (Fig. 1C). Moreover, increased levels of platelet-monocyte aggregates were also documented in dengue patients, especially in patients with thrombocytopenia and increased vascular permeability (Hottz et al., 2014). Ojha et al. (2017) had shown earlier that DENV-2 triggered the platelet activation *in-vitro* in a dose-dependent manner by elevating the expression of P-selectin and PAC-1 binding (Ojha et al., 2017). Moreover, both P-selectin and PAC-1 binding were used as a marker for fibrinogen-induced platelet activation during storage (Hosseini et al., 2019 Oct). However, an increased expression of P-selectin was strongly correlated with a reduction of platelet spreading on the fibrinogen matrix and integrin activation (Hosseini et al., 2019 Oct).

Excessive platelet activation promotes platelet-leukocyte interactions that lead to inflammation and thrombotic events during viral infection (Boilard et al., 2014 May 1; Sung et al., 2019 Jun 3; Hottz et al., 2022 Sep 13; Ghasemzadeh et al., 2022 May). In the case of dengue infection, it has been shown that DC-SIGN and CLEC-2 receptors recognize DENV and activate the inflammatory response on interactions with neutrophils and macrophages (Simon et al., 2015 Jul 16; Sung et al., 2019 Jun 3). Even, the NS1 protein of DENV has been shown to promote inflammation via binding to platelet TLR4 and induces the secretion of IL-1β through the assembly of NLRP3 inflammasome (Quirino-Teixeira et al., 2020 May 12). In response to SARS-CoV-2 infection, platelets recognize the virus through ACE2 and CD147 receptors, activate TLR signaling pathways that lead to enhanced expression of P-selectin and CD40 L, increased platelet-leukocyte interactions, and PLAs formation (Ghasemzadeh et al., 2022 May). Upon activation, platelets release soluble CD40 L (sCD154) in plasma (Henn et al., 1998). Activated platelets have been suggested to secrete approximately 95 % of all sCD154 in blood circulation (André et al., 2002). We demonstrated an elevated expression of membrane-associated CD154 in dengue patients as compared to healthy subjects (Fig. 1E). CD40 L can be found in platelet α-granules and CD40 L expression on activated platelets interacts with CD40 receptors present in immune cells, such as dendritic cells, B cells, and CD8+ *T* cells. This interaction in turn plays a critical role in dendritic cell maturation, B-cell isotype switching, and CD8+ *T* cell response (Elzey et al., 2011 Mar). COVID-19 patients had the highest plasma levels of PF4, serotonin, and sCD40 L released from platelets (Zaid et al., 2020 Sep 17). Platelets derived from COVID-19 patients produce IL-1β, and sCD40 L when stimulated with thrombin suggesting that platelets contribute to inflammation in COVID-19 pathogenesis (Zaid et al., 2020 Sep 17). Several studies have shown the increased expression of platelet-associated CD154 in disease pathogenesis. Expression levels of both membrane-associated and soluble CD154 were significantly higher in platelets of patients with inflammatory bowel disease than in healthy subjects (Danese et al., 2003). HIV-induced release of sCD154 from activated platelets was shown to promote blood-brain barrier permeability in an *in-vivo* mouse model (Davidson et al., 2012). An elegant study in atherosclerotic vascular disease signified the role of platelet-specific CD40 L in thrombus formation using a conditional gene-deficient mice model (Lacy et al., 2021). Additionally, impairment of CD154 signaling has also been attributed to defective thrombotic events and endothelial dysfunction in a mouse model (Hausding et al., 2013). All these findings suggest that CD154 plays a critical role in both inflammation and thrombosis.

Gene enrichment analysis demonstrated that genes involved in the antiviral response, platelet activation, and prothrombotic response were activated during virus infection. Platelet transcriptomes were altered during dengue, influenza, and SARS-CoV-2 infection (Campbell et al., 2019 May 9; Garma et al., 2022 Apr 27). In COVID-19 patients, one of the genes significantly upregulated was CD40LG which codes for CD40 L or CD154 (Garma et al., 2022 Apr 27). In the Malaysian dengue cohort, RNA sequencing of PBMC samples identified a unique set of genes (CCL7, TNFSF15, MYLK2, and SERPINB2) involved with platelet aggregation, inflammation, vascular leakage, and thrombosis as indicators of disease severity in dengue infection (Suppiah et al., 2024 Oct 14). Meta-analysis of publicly available DNA microarray datasets of DENV-infected patients revealed that genes associated with IFN-signaling pathways were upregulated and genes associated with cell-cell interactions were downregulated in DENV infection (Afroz et al., 2016 Sep 21).

We noted a strong positive correlation between CD148 and CD41/CD61 (Fig. 2E) suggesting that both molecules may play a signaling role in the platelet adhesion signaling pathway. CD148 plays a pivotal role in GPVI functional activity. Optimal platelet adhesion and spreading on collagen is dependent on GPVI-mediated inside-out signaling (Hosseini et al., 2020 Aug 31). CD148 dephosphorylates the activation loop tyrosine, thus negatively regulating SFK activity in platelet-specific CD148 knockout mice. CD148-deficient mouse platelets exhibited normal hemostasis, reduced spreading on collagen, and reduced thrombosis (Boulaftali et al., 2018; Zhu, 2018 Mar 8). In hereditary thrombocytopenia, loss of CD148 function resulted in impaired αIIbβ3 signaling and lower pro-platelet production (Mori et al., 2012). Targeted deletion of CD148 also caused inherited thrombocytopenia with bleeding, improper platelet spreading in mouse models, and improper platelet response to agonists and smaller-sized platelets in humans (Senis et al., 2009; Marconi et al., 2019). Altogether, these studies indicate that reduced expression of CD148 may hamper platelet normal function in dengue patients. In line with these observations, we report lower expressions of CD148 as well as CD41/CD61 in dengue patients with platelet counts below 50,000/µL. It has been shown earlier that dengue patients with lower platelet counts exhibited hemorrhagic manifestations (Vedpathak et al., 2024). It is likely that the reduction in CD148 in platelets induces thrombocytopenia and could be associated with increased bleeding tendencies in dengue patients.

Our study has two limitations. Firstly, the sample size, especially the number of healthy subjects and severe dengue patients was small. Secondly, we did not collect follow-up samples to understand the kinetics of hematological, and clinical parameters during the disease.

In summary, we reported the altered expression levels of platelet adhesion molecules in dengue patients. Enhanced expression of CD154 on platelets may stimulate endothelial cells to induce inflammatory response in dengue infection. In contrast, reduced expression of CD148 may enhance bleeding manifestation in dengue patients. However, the role of CD154 and CD148 linked with immunopathogenic events of dengue such as platelet signaling and inflammatory pathways, vascular injury, and hemorrhagic or thrombotic episodes remains to be explored in future studies.

## CRediT authorship contribution statement

**Sayali Vedpathak:** Writing – original draft, Software, Methodology, Formal analysis, Data curation, Conceptualization. **Sonali Palkar:** Resources, Methodology. **AkhileshChandra Mishra:** Writing – review & editing. **Vidya A Arankalle:** Writing – review & editing. **Shubham Shrivastava:** Writing – review & editing, Supervision, Funding acquisition, Formal analysis, Conceptualization.

## Declaration of competing interest

The authors declare that they have no known competing financial interests or personal relationships that could have appeared to influence the work reported in this paper.
